# Comparative Pharmacokinetics of Geniposidic Acid, Genipin-1-*β*-Gentiobioside, Geniposide, Genipin, and Crocetin in Rats after Oral Administration of Crude *Gardeniae Fructus* and Its Three Processed Products Using LC-MS/MS

**DOI:** 10.1155/2020/1642761

**Published:** 2020-05-06

**Authors:** Xuejing Yang, Jin Li, Xi Yang, Jun He, Yan-Xu Chang

**Affiliations:** ^1^School of Pharmacy, Harbin University of Commerce, Harbin, Heilongjiang 150076, China; ^2^Tianjin State Key Laboratory of Modern Chinese Medicine, Tianjin University of Traditional Chinese Medicine, 300193 Tianjin, China; ^3^Tianjin Key Laboratory of Phytochemistry and Pharmaceutical Analysis, Tianjin University of Traditional Chinese Medicine, 300193 Tianjin, China

## Abstract

The extract of *Gardeniae Fructus* (GF) with different processing methods processed the different medicinal properties and efficacy. Crude GF (CGF) could be processed into stir-frying GF (SGF), gancao mix-frying GF (GCGF), and ginger mix-frying GF (GIGF) in practice. An LC-MS/MS method was established for simultaneous quantification of geniposidic acid, geniposide, genipin-1-*β*-gentiobioside, genipin, and crocetin in the rat plasma. The LLOQs for determination of all five components were 10 ng/mL. The accuracies of intraday and interday were in the range of 91%–105%. The recoveries of 5 analytes ranged from 81.0% to 114% with RSD less than 14%. The results showed that the AUCs (area under the plasma concentration-time curve) and *C*_max_ (maximum plasma concentration) of geniposidic acid, genipin-1-*β*-gentiobioside, and geniposide after oral administration of the CGF extract were apparently higher than those after oral administration of other processed extracts. *C*_max_ of geniposide in plasma after administration of GIGF significantly decreased (*p* < 0.01). Genipin was not detected in rat plasma after administration of the GIGF extract, but it can be detected in plasma after administration of CGF, SGF, and GCGF extract. Furthermore, crocin I and crocin II were not detected in plasma samples. Crocetin had higher concentration in rat plasma versus lower contents in extract. It was demonstrated that the different processing methods might influence the pharmacokinetics of geniposidic acid, genipin-1-*β*-gentiobioside, geniposide, genipin, and crocetin.

## 1. Introduction

According to processing theory of traditional Chinese medicine (TCM), medicinal herb needs to be processed in order to improve the efficacy and reduce the toxicity to humans. The medicinal properties of some herb extracts also could be changed to meet the diverse therapeutics acquirements. In China, it is well known that the processing technologies are the characteristic of TCM, and many herbs must be processed before they can be used in clinical prescription.

*Gardeniae Fructus* (Fruits of *Gardenia jasminoides* Ellis, Chinese name “Zhizi,” GF), a frequently used TCM herb, has been broadly applied in clinical use and food additives in China and other Asian countries. GF extract has cold nature and bitter taste, the function of dispelling hot and eliminating dampness, cooling blood, and healing poison, alleviating pain according to the TCM theory [1]. GF extract could be used to treat various diseases, such as febrile diseases, jaundice, acute conjunctivitis, haematuria, epistaxis, and pyogenic infections [2–5]. The extract (*Gardenia* yellow) was widely used as a colorant in food additives [6]. GF could be processed into many different types of products including crude GF (CGF), stir-frying GF (SGF), gancao mix-frying GF (GCGF), ginger mix-frying GF (GIGF), charred GF, and carbonized GF in clinical application [7]. These types of GF extract have usually a common function while each product possesses its individual efficacy characteristics. For example, CGF and SGF extract could be used to dispel hot and GIGF extract could be used to stop vomiting. Meanwhile, the charred GF extract could be used to cool blood and carbonized GF extract could be used to stop bleeding [8].

Lots of studies have being focused on the chemical ingredients in GF. Some GF ingredients have been isolated and identified such as geniposides, geniposidic acid, genipin-1-*β*-gentiobioside, gardenoside, shanzhiside, scandoside methyl ester, methyl deacetyl, deacetyl-asperulosidic acid methyl ester, crocin I, and crocin II [9,10]. It was reported that iridoids have antidepression [11,12], antimicrobial activity [13], anti-inflammatory [14,15], and antitumor [16] effects, while crocins process the hepatoprotection [17], antihyperlipidemic [18], and antidiabetics [19], besides antidepression [20,21], anti-inflammatory [22], and antitumor [19] effects. Pharmacokinetics is an indispensable strategy to know drug behaviors *in vivo* after administration. Pharmacokinetic profiles of multiple components are beneficial for evaluating the safety and efficacy of herbal medicines [23]. Up to now, the pharmacokinetics of some active compounds in GF extract were studied by HPLC-UV [24,25] and HPLC-MS [26–28] analytical method. However, those pharmacokinetics studies of GF extract mainly focus on iridoid glycosides *in vivo*. To the best of our knowledge, there were no reports on the simultaneous pharmacokinetics of iridoids and crocin. In addition, there are no reports about the differences in pharmacokinetic parameters of bioactive compounds between after oral administration of the crude GFs and after other different processed GF extract.

In the present study, a rapid and sensitive HPLC-MS method was developed and validated for simultaneous determination of iridoids and crocins in rat plasma after oral administration of the crude and three processed GF aqueous extracts. The purpose of this study is to clarify the differences in pharmacokinetic parameters of bioactive compounds between after oral administration of the crude GF and the processed GF extract. It could also exhibit how processing method changed the pharmacokinetic action of bioactive components *in vivo*.

## 2. Materials and Methods

### 2.1. Materials and Reagents

The herb CGF and gancao (*Glycyrrhizae Radix et Rhizoma*) were obtained from Anguo TCM market (Hebei, China) and were authenticated by Professor Lin Ma (Tianjin University of Traditional Chinese Medicine). The voucher specimens were deposited at the lab of Tianjin University of Traditional Chinese Medicine. Fresh ginger was purchased from Tianjin market. Geniposidic acid, geniposide, genipin-1-*β*-gentiobioside, genipin, crocin I, crocin II, crocetin, and loganin (internal standard; IS) were purchased from the National Institute for the Control of Pharmaceutical and Biological Products (Beijing, China). The purity of all standards was all above 98%.

Methanol, acetonitrile (Merck, Darmstadt, Germany), and formic acid (Tedia Company Inc., Fairfield, USA) were of chromatographic grade. Purified water produced by a Milli-Q Academic ultra-pure water system (Millipore, Milford, MA, USA) was used in all experiments. All other reagents of analytical grade were obtained from Tianjin Concord Science Co. Ltd. (Tianjin, China).

### 2.2. Preparation of *Gardeniae Fructus*

The processing of SGF extract was that CGF material was tossed into a heated pan and stir-fryed until the surface of the GF slightly turns yellow or gives off an aroma. GCGF is stir-frying GF with gancao (*Glycyrrhizae Radix et Rhizoma*) extract solution (70 g gancao extracted with 168 mL water) that soaks into them. GIGF is stir-frying GF with ginger juice (12.5% ginger juice) that soaks into the herb. 600 g CGF, SGF, GCGF, and GIGF were refluxed twice with water (1 : 10, w/v) for 2 h, respectively. The extraction was combined and condensed to 500 mL. The concentrations of the residues were 1.2 g/mL for CGF, SGF, GCGF, and GIGF extract.

### 2.3. Quantitative Determination of Extracts by UHPLC-DAD

Six analytes (geniposidic acid, geniposide, genipin-1-*β*-gentiobioside, crocin I, crocin II, and crocetin) in GF extracts were quantitatively analyzed. Two grams of sample power (CGF, SGF, GCGF, and GIGF) was extracted twice with water (1 : 10, w/v) under reflux in a water bath for 2 h, respectively. After the extracts were filtered and combined, the solution was evaporated to dryness. The yields of CGF, SGF, GCGF, and GIGF extract were 32.99%, 35.82%, 35.8%, and 36.13%, respectively.

The extract (20 mg) was precisely weighted and dissolved with methanol (10 mL) under ultrasonically for 30 min. After replenished with methanol by weight loss, the extraction solution was diluted twice and filtered through a 0.22 membrane filter. The analyses were performed by UPLC-PDA method according to our previous publication [29].

### 2.4. Quantitative Determination of Plasma Sample by LC-ESI-MS/MS

The LC-ESI-MS/MS system is composed of an Agilent 1200 series LC system (Agilent Technologies, USA) including a binary pump, a vacuum degasser unit, an autosampler, and an API 3200 triple quadrupole mass Spectrometer with an ESI source (Concord, Ontario, Canada). Data was acquired by Analyst 1.4.2 software (AB MDS Sciex).

The Agilent Eclipse plus C_18_ (4.6 × 100 mm, 1.8 *μ*m) column with a guard column was used to separate multiple ingredients. The mobile phases were methanol (A) and water (B) with a gradient elution of 3–36% A at 0–3 min, 36–36% A at 3–15 min, 36–97% A at 15–16 min, and 97–97% A at 16–26 min, then back from 97% to 3% balanced for 10 min. The flow rate was set at 0.3 mL/min and the column temperature was set at 30°C. The injection volume was 5 *μ*L.

The detection was operated in Multiple Reaction Monitoring (MRM) mode. The source parameters were as follows: curtain gas, collision gas, ion spray voltage, temperature, ion source gas 1, and ion source gas 2 were set at 20 psi, 5 psi, 5500 V, 450°C, 40 psi, and 40 psi, respectively. Nitrogen was the only gas used in the experiment. The other parameters are listed in Table 1.

### 2.5. Preparation Standard Solution and Quality Control Samples

The appropriate amounts of geniposidic acid, genipin-1-*β*-gentiobioside, geniposide, genipin, and crocetin were separately weighted and prepared in methanol as stock solutions. The IS stock solution of loganin was also prepared in methanol and kept at 100 ng·mL^−1^ level in each working solution and sample. The stock solutions were prepared together as a mixed standard solution, and then it was gradually diluted into a series of concentrations as mixed working solution. All the working solutions were stored at 4°C before use.

Quality control (QC) samples containing geniposidic acid, geniposide, genipin-1-*β*-gentiobioside, genipin, and crocetin were prepared at LLOQ (10 ng/mL), low (30 ng/mL), medium (300 ng/mL), and high concentrations (3000 ng/mL), spiking with appropriate standard solutions with blank plasma to establish calibration curve and method validation. The samples were prepared by the same procedures for plasma samples as described below.

### 2.6. Animal Experiment and Plasma Sample Preparation

A total of 24 male Sprague-Dawley rats (weight: 220–250 g) were obtained from animal center of Tianjin University of Traditional Chinese Medicine (Tianjin, China). Those rats were randomly divided into four groups (CGF, SGF, GCGF, and GIGF groups) and were housed in an environmentally controlled room (temperature 20–25°C, humidity 60 ± 5%). The rats were allowed free accessibility to food and water in the first week. They were fasted for 12 h before the experiment with water taken freely. The animal experiments were according to the Guidelines for the Care and Use of Laboratory Animals and were approved by the Animal Ethics Committee of Tianjin University of Traditional Chinese Medicine. The rats were randomly divided into four groups. Six rats in each group were separately given single dose of 0.75 mL/kg (0.9 g·kg herbal medicine) extract solution of four kinds of GFs vial oral administration. Blood samples (about 300 *μ*L) were collected at 0, 0.08, 0.25, 0.5, 1, 2, 4, 6, 8, 10, 12, 14, and 24 h. The blood samples were collected in heparinized tubes and immediately centrifuged at 6000 rpm for 10 min at 4°C. The plasma samples were stored at −80°C until analysis.

The plasma sample (100 *μ*L) was spiked with 20 *μ*L IS working solution and 10 *μ*L formic acid in an Eppendorf tube. Samples were vortex for 30 s, and then 1 mL acetonitrile was added to precipitate protein. Then samples were vortexed for 2 min and the tubes were centrifuged at 14000 rpm for 10 min. The upper organic phase was transferred into a new tube and evaporated to dryness under a flow of nitrogen gas. The residue was reconstituted with 50% methanol 100 *μ*L. After centrifuging at 14000 rpm for 10 min, the supernatant was transferred into an autosampler vial and a volume of 5 *μ*L was injected into the HPLC-ESI-MS/MS for analysis.

### 2.7. Method Validation

The method was validated in terms of specificity, linearity, accuracy and precision, recovery, matrix effect, and stability according to the USA Food and Drug Administration (FDA) bioanalytical method validation guidelines.

#### 2.7.1. Method Validation Linearity

The calibration curves were constructed to calculate the linearity by the plot of the peak area ratios of analytes versus the IS against the concentrations using a 1/*X*^2^ weighted linear least-squares regression model. The lower limit of quantification (LLOQ) of the assay was defined as the lowest concentration of the standard curve at which the signal-to-noise ratio (*S/N*) was preliminary found to be larger than 5. It was acceptable that the precision was less than 20% and the accuracy was within ±20%.

#### 2.7.2. Specificity

Blank plasma samples were obtained from six individual rats. The blank plasma, blank plasma spiked with mixed standard solution, and the real plasma samples were compared by their chromatographic profiles to exclude endogenous interference.

#### 2.7.3. Precision and Accuracy

The precision and accuracy were assessed by analyzing QC samples at LLOQ, low, medium, and high concentrations. The intraday precision and accuracy were evaluated by six-replicate quality control samples in the same day. The interday precision and accuracy were evaluated by six-replicate samples on three consecutive days. The precision was expressed by the relative standard deviation (RSD).

#### 2.7.4. Extraction Recovery and Matrix Effect

The extraction recoveries of 5 ingredients from GF were determined at four QC levels with six replicates. It was calculated by comparing the peak areas ratios of the processed samples with those of postprocessed spiked samples. The matrix effects were calculated by comparing the peak areas ratios of the analytes in postprocessed spiked samples with those of the analytes in pure standard solution.

#### 2.7.5. Stability

The stability of 5 components in the plasma was obtained by evaluating QC samples at four concentration levels with three replicates in different conditions. The QC samples were kept at room temperature for 12 h to determine the short-term stability. Freeze-thaws stability was tested after three freeze-thaw (−80°C at room temperature) on three consecutive days. Long-term stability was tested by evaluating QC samples stored at −80°C for one month.

### 2.8. Pharmacokinetics Study and Statistical Analysis

The pharmacokinetic parameters were calculated using a Drug and Statistics 1.0 (DAS 1.0) software (Medical College of Wannan, China). The pharmacokinetic parameters included time of maximum concentration (*T*_max_), maximum plasma concentration (*C*_max_), elimination half-life (*T*_1/2_), and area under concentrations curve (AUC_0–t_ and AUC_0–∞_). All values were expressed as mean ± SD. A paired *t* test was used to test the difference among these groups.

## 3. Results and Discussion

### 3.1. The Content of the Six Analytes in GFs Extracts

The original content of compounds in herb extract was a nonnegligible factor to influence the pharmacokinetics results. The contents of geniposidic acid, genipin-1-*β*-gentiobioside, geniposide, crocin I, crocin II, and crocetin are 0.449, 1.48, 5.374, 0.327, 0.058, and 0.014% in CGF extract; 0.429, 1.574, 5.079, 0.403, 0.066, and 0.016% in SGF extract; 0.421, 1.488, 5.058, 0.384, 0.064, and 0.017% in GCGF extract; 0.414, 1.598, 5.241, 0.377, 0.066, and 0.015% in GIGF extract, respectively. Compared with CGF extract, the contents of geniposidic acid and geniposide in the three processing products were reduced. On the contrary, the contents of genipin-1-*β*-gentiobioside were increased in processed ones with different processed method. For all three crocin compounds, the contents of crocin I, crocin II, and crocetin in the three processed GF extracts were all higher than those in CGF. These results would contribute to the analysis of the exposure level in blood and make the pharmacokinetics study more objective and reasonable.

### 3.2. Optimization of Method Condition

To achieve better resolution and good peak shape, the chromatographic conditions were optimized by using methanol, acetonitrile, water, and water with different proportions of formic acid. As a result, acetonitrile-water was chosen as the mobile phase to obtain high response intensity and good peak shape for the five analytes. Both positive and negative detection modes were compared to get better mass response of analytes. Negative ionization mode was chosen and MRM mode was applied due to its higher sensitivity. The related mass parameters of all five analytes and IS were optimized to obtain better ionization efficiency (Table 1).

Liquid-liquid extraction (LLE) and protein precipitation (PPT) methods were examined to extract the iridoids and crocins from plasma samples. Ethyl acetate containing different concentrations of formic acid was estimated as LLE solvents. Methanol, acetonitrile, and acetonitrile added different concentrations of formic acid were tested for PPT. It was found that acetonitrile and formic acid were optimized as the pretreatment method of plasma samples according to negligible matrix effect and high extraction recovery for all analytes.

### 3.3. Method Validation

#### 3.3.1. Specificity

**Figure 1:**
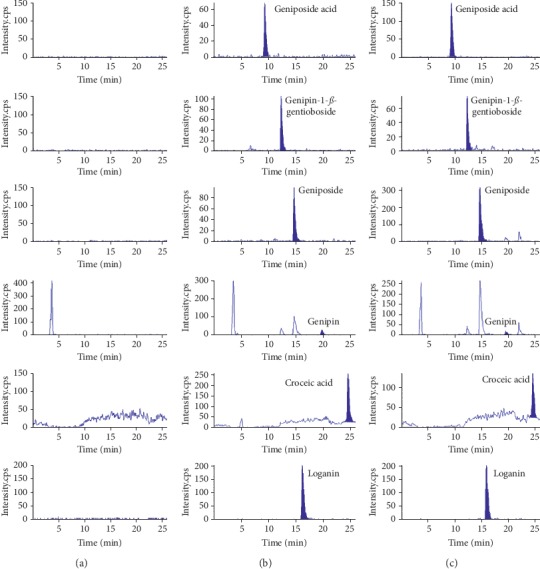
MRM chromatograms of all components in rat plasma: (a) blank plasma; (b) blank plasma spiked with five analytes at LLOQ (10 ng/mL) and IS; (c) the plasma sample at 0.5 h after oral administration of CGF.

The specificity of LC-MS/MS was evaluated by analyzing blank plasma samples, blank plasma spiked with mixed standards, and plasma obtained after oral administration for 30 min of GF. As shown in Figure 1, there were no interferences and endogenous interference at their peak region in the chromatogram profile.

#### 3.3.2. Linearity and LLOQ

The linear range was from 10 to 3000 ng/mL for the five components. Good linearity was determined in the validation concentration range (all correlation coefficients > 0.994). The calibration curve equations and correlation coefficients of the ingredients were as follows: *y* = 0.00328*x* + 0.0115, *r* = 0.9944 for geniposidic acid, *y* = 0.00629*x* + 0.00621, *r* = 0.9976 for genipin-1-*ß*-gentiobioside, *y* = 0.00252*x* + 0.00791, *r* = 0.9961 for geniposide, *y* = 0.00115*x* + 0.00438, *r* = 0.9985 for genipin, and *y* = 0.00341*x* + 0.0397, *r* = 0.9952 for crocetin. The lower limits of quantification (LLOQ) for determination of all five analytes were all 10 ng/mL. These results illustrated that the newly LC-MS/MS method was proper for the quantitative detection.

#### 3.3.3. Accuracy and Precision

The intraday and interday precision and accuracy were determined by replicate analysis of QC samples on the same day (intraday) and continuously for 3 days (interday), respectively. The intraday and interday precision and accuracy are shown in Table 2. The intraday and interday precisions of the analytes met the requirement of method validation, and the accuracies were in the range of 91%–105%. The results demonstrated that the precision and accuracy of the newly LC-MS/MS method were accurate, reliable, reproducible, and acceptable.

#### 3.3.4. Extraction Recovery and Matrix Effect

The mean extraction recoveries of 5 analytes at four different concentrations were ranged from 81.0 ± 7.4% to 114 ± 5% with RSD less than 13.55%. The matrix effect was from 88.6 ± 5.7% to 115 ± 2% with RSD less than 13.47%. The data showed that the extraction recovery and matrix effect of this method were reliable and reproducible. The extraction efficiencies of 5 ingredients were acceptable (Table 3).

#### 3.3.5. Stability

The stability of the five components in rat plasma was determined by evaluating QC samples stored at different temperature and timing conditions. All the components were stable in the autosampler for 24 h, after three freeze-thaw cycles and at a month at −80°C. As listed in Table 4, the results indicated that the five analytes in rat plasma were stable at different storage conditions with an RSD range of 1.03–13.44%.

### 3.4. Pharmacokinetics Study

**Figure 2:**
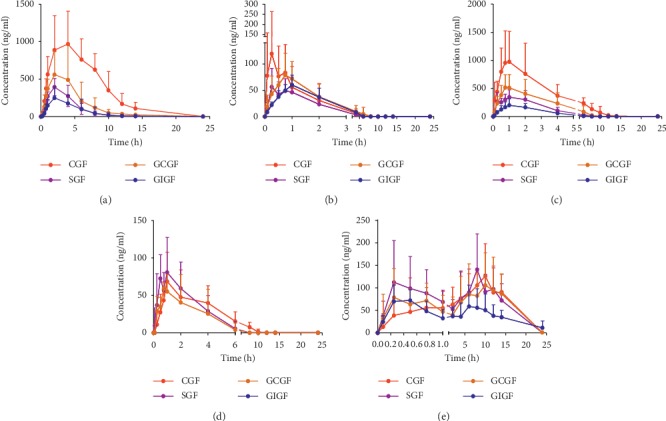
Means pharmacokinetic profiles multiple bioactive components after oral administration of CGF, SGF, GCGF, and GIGF extract to rats (*n* = 6). (a) Geniposidic acid, (b) genipin-1-*β*-gentiobioside, (c) geniposide, (d) genipin, and (e) crocetin.

The newly established LC-MS/MS method was validated and applied to pharmacokinetics studies of five bioactive components in the plasma after oral administration of CGF, SGF, GCGF, and GIGF aqueous extracts to rats. Five components were distributed as an opened single-compartment model, and the mean pharmacokinetic profiles are presented in Figure 2. Meanwhile, the pharmacokinetic parameters including AUC_(0–t24)_, AUC_(0–∞)_, *C*_max_, *T*_1/2_, and *T*_max_ are summarized in Table 5.

After oral administration of CGF extract, *T*_max_ was ranged from 0.55 to 3.33 h and *T*_1/2_ was ranged from 0.72 to 3.42 h for four iridoids while *T*_max_ was 7.30 ± 3.93 h and *T*_1/2_ was 41.65 ± 57.62 h for crocetin. It was indicated that iridoids exhibited more rapid absorption than crocins in GF extract. Geniposidic acid, which had lower content than genipin-1-*ß*-gentiobioside and geniposide, presented a higher exposure level. The values of AUC_(0–24 h)_ and AUC_(0–∞)_ of geniposidic acid were obviously higher than those of other compounds. Geniposide, which was the highest content in GF extracts, showed a lower exposure level than geniposidic acid. Genipin could be hardly determined in GF extracts, but it could be detected in plasma samples after oral administration of GF extract. This phenomenon might be related to the fact that geniposide was hydrolyzed into genipin with the help of intestinal flora [24]. Crocin I and crocin II were determined in GF extract, but they could not be determined in rat plasma after oral administration of GF extract. Crocetin, whose content was lower than those of crocin I and crocin II in GF extract, showed a relatively higher blood exposure level. The reason may be that crocin was transformed into crocetin quickly in the gastrointestinal tract after oral administration of crocin, and the exposure of its metabolite, crocetin, was much higher than crocin [30].

Regarding CGF extract as a control group, *C*_max_ (ng·mL^−1^), AUC_(0–24 h)_ (ng/L·h), and AUC_(0–∞)_ (ng/L·h) of geniposidic acid and geniposide of SGF-, GCGF and GIGF extract groups were significant (*p* < 0.05). The values of these three parameters of geniposidic acid and geniposide in plasma after intragastric administration of three processed extracts were significantly decreased. Those three parameters of genipin-1-*β*-gentiobioside, genipin, and crocetin of SGF- and GCGF-treated group were not significantly different. *T*_max_ of genipin-1-*β*-gentiobioside and crocetin were significantly different. Setting SGF extract treated group as a control group, *C*_max_ of geniposide was significantly decreased comparing with GCGF extract-treated and GIGF extract-treated groups. This result indicated that ginger mix-frying could reduce the bioavailability of geniposide. It was worth noticing that the absorption of geniposidic acid in rat plasma was decreased sharply after oral administration of processed GFs (AUC_(0–t24),_ CGF : SGF : GCGF : GIGF, 1 : 0.22 : 0.39 : 0.15), while the content of geniposidic acid in herbal extracts declined very slightly after processing (content, CGF : SGF : GCGF : GIGF, 1 : 0.94 : 0.74 : 0.76). It was indicated that the processing method could reduce the absorption of geniposidic acid *in vivo*. *T*_max_ and AUC_(0–24 h)_ parameters of crocetin in GIGF extract were significantly different (*p* < 0.05).

Genipin could be hardly determined in GF extract and it could be detected in plasma after oral CGF, SGF, and GCGF extract as a metabolite of geniposide. Genipin could not be detected in GIGF extract, which was probably caused by the inhibition of absorption after ginger mix-frying. Interestingly, as for genipin-1-*β*-gentiobioside, an additional small peak was observed before the maximum plasma concentration in CGF- and SGF-treated groups, and one peak only appeared in GCGF- and GIGF-treated groups (Figure 2(b)). The same inconsistent phenomenon also emerged in crocetin as shown in Figure 2(e), double-peak phenomenon was found in SGF-, GCGF-, and GIGF-treated groups, while one-peak phenomenon was observed in CGF-treated group. Their *T*_max_ also occurred at different times; thus, it was at 1.29 ± 2.31 h for the GIGF-treated group and 6.43–6.61 h for SGF- and GCGF-treated groups. The reasons for all those results need further detailed investigation.

## 4. Conclusion

A rapid, sensitive, and stable LC-MS/MS method was established for simultaneous quantification of geniposidic acid, geniposide, genipin-1-*β*-gentiobioside, genipin, and crocetin in rat plasma. The method validation was successfully been used in the pharmacokinetics study of five components after oral administration of the different GF extract. The results exhibited that different processing methods could obviously affect the absorption of geniposidic acid, geniposide, genipin-1-*β*-gentiobioside, genipin, and crocetin in rats. The differences of pharmacokinetic parameter were probably induced by the processing progress affecting the content and inhibiting the absorption of their two respects. In the future, the intensive study that processing inhibited absorption of geniposide and then changed the production of genipin is necessary, to prove the processing progress changed the physiological disposition and metabolic profile of the components. Further research on metabolic profile change of crocin I and crocin II is also needed.

## Figures and Tables

**Table 1:** MRM scanning and mass spectrometry parameters of analytes and IS.

Compounds	Parameters						
	Q1	Q3	DP	EP	CE	CXP	Rt.(min)
Geniposidic acid	373.0	123.2	−35	−6	−27	−2	9.33
Genipin-1-*β*-gentiobioside	548.9	224.8	−57	−7	−22	−5	12.29
Geniposide	386.8	224.9	−37	−3	−15	−2	14.72
Genipin	224.7	101.1	−54	−3	−17	−4	19.53
Crocetin	327.0	283.0	−28	−7	−18	−2	24.64
Loganin (IS)	389.2	227.1	−26	−3	−15	−4	15.8

**Table 2:** Intraday and interday precision and accuracy of 5 compounds (*n* = 6).

Compounds	Concentration (ng/mL)	Intraday		Interday	
		RSD (%)	Accuracy (%)	RSD (%)	Accuracy (%)
Geniposidic acid	10	4.94	104	2.62	101
	30	3.83	99.5	0.55	99.6
	300	5.55	105	3.19	101
	3000	3.58	100	2.61	97

Genipin-1-*β*-gentiobioside	10	5.29	102	0.79	101
	30	3.63	99	5.20	99
	300	4.50	93	4.10	98.9
	3000	2.04	98	3.03	98.2

Geniposide	10	3.66	96.5	4.51	97.7
	30	4.67	98	3.54	95
	300	5.03	94	3.15	96.6
	3000	3.68	93	0.56	93.9

Genipin	10	7.35	96.5	2.20	98.7
	30	9.32	95	3.19	99
	300	3.61	91	1.71	92.9
	3000	4.14	94.5	2.43	91.3

Crocetin	10	8.84	100	2.08	97.4
	30	4.71	98	0.56	98.6
	300	3.66	97.5	1.32	96.7
	3000	8.37	96	1.14	97.1

**Table 3:** Recoveries and matrix effects of 5 compounds (*n* = 3).

Compounds	Concentration (ng/mL)	Recovery		Matrix effect	
		Mean ± SD (%)	RSD (%)	Mean ± SD (%)	RSD (%)
Geniposidic acid	10	96.7 ± 4.8	4.96	114 ± 10	8.77
	30	110 ± 13	11.82	103 ± 10	9.71
	300	111 ± 6	5.41	110 ± 4	3.64
	3000	97.8 ± 4.1	4.19	115 ± 2	1.74

Genipin-1-*β*-gentiobioside	10	97.2 ± 5.5	5.66	92.5 ± 7.5	8.11
	30	94.7 ± 2.1	2.22	114 ± 4	3.51
	300	100 ± 12	12.01	88.6 ± 5.7	6.43
	3000	94.8 ± 4.1	4.32	93.9 ± 4.7	5.01

Geniposide	10	97.4 ± 9.4	9.65	97.3 ± 11.5	11.82
	30	110 ± 4	3.64	96.4 ± 3.7	3.84
	300	84.3 ± 4.0	4.74	106 ± 6.6	6.23
	3000	104 ± 3	2.88	111 ± 6	5.41

Genipin	10	81.2 ± 11.0	13.55	106 ± 9	8.49
	30	81.0 ± 7.4	9.14	115 ± 2	1.74
	300	111 ± 11	9.91	104 ± 9	8.65
	3000	114 ± 5	4.39	92.9 ± 5.2	5.60

Crocetin	10	103 ± 8.	7.77	89.1 ± 4.2	4.71
	30	95.8 ± 10.0	10.44	94.3 ± 6.8	7.21
	300	99.2 ± 13.4	13.51	98.0 ± 13.2	13.47
	3000	98.5 ± 10.0	10.15	98.3 ± 5.4	5.49

**Table 4:** Stability of the 5 compounds (*n* = 6).

Compounds	Concentration (ng/mL)	Freeze-thaw cycles		At −80°C for a month		Autosampler for 24 h	
		RSD (%)	Remain (%)	RSD (%)	Remain (%)	RSD (%)	Remain (%)
Geniposidic acid	10	10.37	96.3	7.48	107	1.42	94.7
	30	5.71	101	10.14	98.0	6.05	96.4
	300	6.44	107	5.30	106	3.40	96.4
	3000	2.43	108	1.55	99.9	3.94	96.7

Genipin-1-*β*-gentiobioside	10	3.79	104	13.44	95.8	10.40	99.3
	30	1.03	105	10.50	96.3	1.52	100
	300	7.71	101	4.19	95.1	5.96	101
	3000	2.60	103	0.87	95.1	2.75	90.7

Geniposide	10	4.10	97.5	10.75	99.2	10.57	102
	30	5.17	92.8	9.85	98.4	4.11	95.1
	300	1.88	93.1	2.79	94.8	7.91	96.3
	3000	5.92	95.0	2.97	92.8	1.24	91.6

Genipin	10	3.81	101.	3.14	108	3.24	95.4
	30	4.43	95.3	12.68	103	4.49	104
	300	2.68	95.9	5.98	105	0.76	90.6
	3000	9.22	100	6.99	101	4.12	92.6

Crocetin	10	2.02	102	9.75	98.4	6.43	97.9
	30	8.05	95.3	10.15	101	6.00	99.9
	300	7.02	98.6	6.67	105	4.41	96.3
	3000	6.52	106	1.33	103	5.55	98.9

**Table 5:** The main pharmacokinetics of 5 compounds in rat plasma (*n* = 6).

Compounds	Treatment	*C*_max_ (ng/mL)	*T*_max_ (h)	*T*_1/2_ (h)	AUC_(0–24 h)_ (ng/L·h)	AUC_(0–∞)_ (ng/L·h)
Geniposidic acid	CGF	1022.83 ± 450.17	3.33 ± 1.63	2.52 ± 0.64	7696.99 ± 2741.28	8586.63 ± 3450.88
	SGF	398.33 ± 107.8^*∗∗*^	1.78 ± 0.44^*∗*^	2.93 ± 3.45	1730.86 ± 618.74^*∗∗*^	1774.38 ± 654.94^*∗∗*^
	GCGF	669.63 ± 431.89	3.33 ± 3.43	1.72 ± 0.69^*∗*^	2988.13 ± 2302.06^*∗∗*^	3250.09 ± 2342^*∗∗*^
	GIGF	272.00 ± 164.04^*∗∗*^	2.40 ± 1.26	1.50 ± 0.42^*∗∗*^	1187.21 ± 813.41^*∗∗*^	1351.01 ± 827.12^*∗∗*^

Genipin-1-*β*-gentiobioside	CGF	149.90 ± 132.07	0.55 ± 0.33	0.72 ± 0.25	141.12 ± 104.11	237.29 ± 119.51
	SGF	79.08 ± 39.47	0.61 ± 0.31	0.61 ± 0.18	109.26 ± 58.78	121.03 ± 54.86^*∗*^
	GCGF	102.92 ± 58.15	0.78 ± 0.20	0.78 ± 0.32	206.27 ± 142.55	233.30 ± 165.74
	GIGF	62.94 ± 16.81	1.05 ± 0.37^*∗*#^	0.91 ± 0.36^#^	131.30 ± 36.78	151.67 ± 48.73

Geniposide	CGF	1071.67 ± 598.46	1.54 ± 1.30	3.21 ± 2.31	4000.13 ± 1677.19	4119.45 ± 1649.61
	SGF	455.44 ± 160.36^*∗*^	0.89 ± 0.72	1.61 ± 0.63	1141.92 ± 389.07^*∗∗*^	1200.78 ± 382.09^*∗∗*^
	GCGF	581.00 ± 240.71^*∗*^	0.86 ± 0.13	1.44 ± 0.53^*∗*^	1862.67 ± 1078.69^*∗∗*^	1899.73 ± 1092.04^*∗∗*^
	GIGF	211.50 ± 109.10^*∗∗*##^	1.30 ± 0.48	1.08 ± 0.45^*∗*^	733.81 ± 519.14^*∗∗*^	781.95 ± 530.75^*∗∗*^

Genipin	CGF	76.44 ± 40.93	1.11 ± 0.40	3.42 ± 1.94	265.30 ± 103.43	356.72 ± 139.94
	SGF	98.05 ± 41.84	1.00 ± 0.55	1.39 ± 0.88	233.77 ± 141.78	281.41 ± 178.60
	GCGF	68.52 ± 19.41	1.10 ± 0.52	1.39 ± 0.98	152.67 ± 113.60	329.06 ± 394.68
	GIGF	—	—	—	—	—

Crocetin	CGF	165.02 ± 52.86	7.30 ± 3.93	41.65 ± 57.62	1251.80 ± 354.98	10824.60 ± 13505.86
	SGF	152.51 ± 81.90	6.61 ± 4.05	56.17 ± 42.99	1258.95 ± 596.55	7510.78 ± 8494.79
	GCGF	162.66 ± 76.43	6.43 ± 5.02	12.75 ± 6.64^#^	1098.06 ± 522.58	3845.62 ± 5252.68
	GIGF	91.18 ± 35.65^*∗*^	1.29 ± 2.31^*∗*#^	31.84 ± 29.27	621.22 ± 389.71^*∗*#^	3610.39 ± 2206.09

CGF as control group, *p* < 0.05^*∗*^; *p* < 0.01^*∗∗*^, SGF as control group, *p* < 0.05^#^; *p* < 0.01^##^.

## Data Availability

The data used to support the findings of this study are included within the article.
